# Case Report: Clinical management of a severe DBA patient with a novel RPS19 mutation

**DOI:** 10.3389/fped.2025.1590183

**Published:** 2025-05-26

**Authors:** Junfen Zhou, Jiayi Zhong, Yisha Zhao, Miaojun Mo, Xianbo Chen, Huangjia Zhou, Luya Zhang, Li Lin, Yichi Zhang, Xiaohong Tao, Xianhua Mao, Haiting Li, Enfu Tao

**Affiliations:** ^1^ Department of Pediatrics, Wenling Maternal and Child Health Care Hospital, Wenling, Zhejiang, China; ^2^ Department of Pharmacy, Wenling Maternal and Child Health Care Hospital, Wenling, Zhejiang, China; ^3^ Department of Neonatology and NICU, Wenling Maternal and Child Health Care Hospital, Wenling, Zhejiang, China

**Keywords:** Diamond-Blackfan anemia, severe anemia, shock, ribosomal protein genes, metabolic acidosis, hemodynamic instability, blood transfusion

## Abstract

Diamond-Blackfan anemia (DBA) is a rare congenital bone marrow failure disorder characterized by defective erythropoiesis, typically caused by mutations in ribosomal protein (RP) genes, most commonly *RPS19*. It usually presents in early infancy with severe anemia, growth retardation, and an increased risk of congenital malformations and malignancies. However, cases of DBA leading to severe anemia and shock are exceedingly rare. This case report describes a life-threatening presentation of DBA in a 56-day-old female infant who presented with severe anemia and shock. The infant was admitted with a 2-day history of poor feeding and persistent crying, accompanied by hypothermia (34.4°C), unresponsiveness, and profound pallor. Initial laboratory findings revealed critical anemia (hemoglobin 18 g/L) and severe metabolic acidosis (pH 6.61, base excess −36.06 mmol/L). Hemodynamic instability, including undetectable blood pressure and prolonged capillary refill time, indicated shock. Immediate interventions, including volume expansion with normal saline, correction of acidosis with sodium bicarbonate, and packed red blood cells (PRBCs) transfusion, stabilized the infant. Genetic testing identified a *de novo* heterozygous mutation in the *RPS19* gene (c.3G > T), confirming the diagnosis of DBA. Over the course of a 1-year follow-up, the infant required regular blood transfusions at approximately 4-week intervals to sustain hemoglobin levels within the range of 69–86 g/L. Growth retardation and poor appetite were observed, consistent with the known complications of DBA. This case highlights the importance of early recognition and aggressive management of severe anemia in infants, particularly in the context of DBA, to prevent life-threatening complications such as shock and metabolic acidosis. The role of genetic testing in confirming the diagnosis and guiding long-term management is emphasized. This report also reviews the literature on DBA, focusing on the pathophysiology of anemia, the association between *RPS19* mutations and clinical phenotypes, and the challenges of managing transfusion-dependent patients. The findings underscore the need for a multidisciplinary approach to DBA, including regular monitoring for complications such as iron overload, growth retardation, and malignancy risk. Early genetic counseling and tailored therapeutic strategies are crucial for improving outcomes in this rare and complex disorder.

## Introduction

Diamond-Blackfan anemia (DBA), recently reclassified as DBA syndrome, is a rare congenital bone marrow failure disorder characterized by erythroid hypoplasia due to defective erythropoiesis in red blood cell precursors (1, 2). It is typically caused by loss-of function variants in genes encoding ribosomal proteins (RP) (3). The condition usually manifests during early infancy, with 95% of patients diagnosed before the age of 2 years (4). While many patients are asymptomatic carriers, the majority of DBA cases present with severe anemia within the first year of life, requiring continuous treatment (3) with a median age of onset between 2 and 3 months (2, 5, 6). Notably, DBA is associated with significant comorbidities, including an increased risk of congenital malformations (particularly craniofacial and upper limb anomalies), growth retardation, and a predisposition to malignancy (5).

To date, mutations in 24 RP genes have been identified in DBA (7). Among these, *RPS19*, *RPL5*, *RPS26*, and *RPL11* are the most frequently mutated RP genes (8). Notably, *RPS19* is the first and most common mutated gene, accounting for approximately 25% of cases (7) and represents a significant cause of severe anemia in infants (9).

Although severe anemia in infants with DBA has been documented in literature, instances of severe anemia and even shock caused by DBA in infants are rare (8, 10). Moreover, the early prognosis of infants with severe anemia and shock due to DBA has not been reported (10). We present a rare case of a 56-day-old female infant with a *de novo RPS19* mutation, who presented with severe anemia and shock, and subsequently experienced growth retardation during follow-up.

## Case description

A 56-day-old female infant was presented to the emergency department with a 2-day history of poor feeding and half a day of persistent crying. There was no history of hematemesis, melena, or trauma. The family, including parents, grandparents, and a 2-year-old sister, were healthy with no genetic or metabolic disorders.

Upon assessment, the infant showed critical signs: hypothermia (34.4°C), unresponsiveness, labored breathing with groaning, and profound pallor. She was in shock, with undetectable blood pressure and a prolonged capillary refill time of 3 s. Emergent laboratory tests indicated severe anemia and metabolic acidosis, with hemoglobin of 18 g/L, pH of 6.61, and base excess of −36.06 mmol/L. Moreover, blood biochemistry showed indicated elevated total bilirubin (25.2 μmol/L), lactate dehydrogenase (416 IU/L), potassium (6.12 mmol/L), uric acid (742 µmol/L), urea (6.7 mmol/L), creatinine (41 µmol/L), and glucose (16.78 mmol/L), suggesting renal impairment, metabolic abnormalities and electrolyte disturbance. The Coombs test was negative, ruling out immune-related hemolysis. Immediate treatment included two intravenous lines, oxygen via headbox, cardiac monitoring and a radiant warmer. Initial therapy involved IV normal saline (20 ml/kg) for volume expansion, ceftriaxone 200 mg IV as empirical antibiotic therapy, and methylprednisolone sodium succinate at a dose of 2 mg/kg every 12 h IV for anti-inflammatory effects. The patient received further treatment with 2 mg IV furosemide for diuresis, 1.4% sodium bicarbonate infusion to correct metabolic acidosis, and an intramuscular vitamin K1 injection to prevent potential hemorrhagic complications. Furthermore, a transfusion of 20 ml/kg red blood cells (RBC) to relieve anemia. Following successful resuscitation measures, the patient showed significant improvement and then was transferred to the pediatric inpatient unit for further treatment and monitoring.

**Figure 1:**
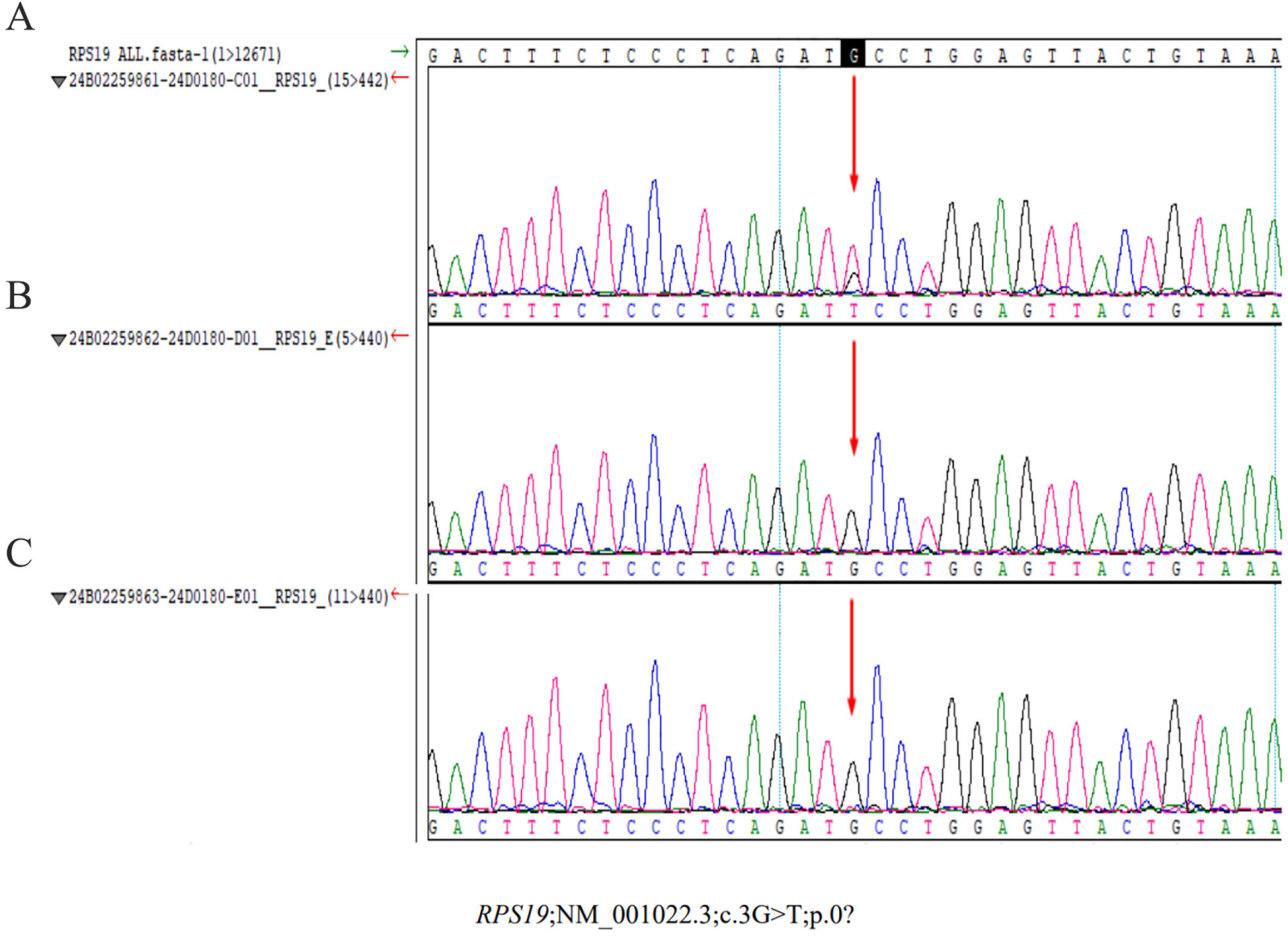
Sanger sequencing results of the *RPS19* gene mutation in the proband and parents. **(A)** Proband. A heterozygous mutation (c.3G > T) was identified in the proband, affecting the translation initiation codon (p.0?). This variant, located in exon 2/coding sequence 1 (EX2/CDS1) of the *RPS19* gene, was absent in both parents, confirming its *de novo* origin. **(B)** (Mother) and **(C)** (father): no *RPS19* gene mutations detected in parental samples.

On admission, the infant presented with hypothermia (35.5°C), tachycardia (141 bpm), a respiratory rate of 44 breaths/min, and blood pressure of 82/29 mmHg. The patient was conscious but lethargic, with dry oral mucosa, pale yellowish complexion, and inspiratory retractions. Physical examination revealed coarse breath sounds without rales, regular heart rhythm without murmurs, and no hepatosplenomegaly. Neurological assessment was unremarkable. Repeated arterial blood gas analysis showed significant improvement in metabolic acidosis with pH of 7.43, base excess of −7.44 mmol/L, although lactate levels remain elevated (lactate of 6.5 mmol/L). The next day's complete blood count showed hemoglobin at 51 g/L, and a reticulocyte percentage of 0.3%, indicating a slight rise in hemoglobin but consistently low reticulocyte count. Further diagnostic tests were conducted. Stool routine tests showed no abnormalities, and the coagulation profile was normal. Urine metabolic screening ruled out inherited metabolic disorders. Bedside chest and abdominal radiographs revealed increased lung markings and an enlargedheart. Abdominal ultrasound found no structural issues, excluding hemorrhagic disorders. Cranial imaging was normal. Echocardiography revealed a 3.1 mm atrial septal defect. The patient was treated with ceftriaxone for 3 days before stopping. Methylprednisolone was administered at 2 mg/kg every 12 h, tapered to 1 mg/kg daily, and stopped after 5 days. Serial laboratory tests showed significant improvement: hemoglobin increase to 74 g/L, renal function, bilirubin and blood glucose were normal. Whole-exome sequencing identified a *de novo* heterozygous mutation in the *RPS19* gene (NM_001022.3; c.3G > T; p.0?; gene subregion: EX2/CDS1, genotype: Heterozygous), which was further confirmed by sanger sequencing (Figure 1A). Parental genetic testing revealed the absence of the *RPS19* mutation in both parents (Figure 1B,C). The final diagnoses included DBA, shock, severe anemia, multiple organ dysfunction syndrome, metabolic acidosis, malnutrition, atrial septal defect, hypothermia, hyperlactatemia, and electrolyte disturbance. The patient was discharged in stable condition after 8 days without complications. The key laboratory findings are presented in Table 1.

**Table 1:** Key laboratory findings at presentation.

Category	Parameter	Value	Reference range
Complete blood count	WBC	15.1 × 10⁹/L	4.3–14.2 × 10⁹/L
	Hemoglobin	18 g/L	97–141 g/L
	Platelets	697 × 10⁹/L	183–614 × 10⁹/L
	Reticulocytes	0.3%	0.5%–1.5%
	CRP	1.5 mg/L	<0.5 mg/L
Blood Gas	pH	6.61	7.35–7.45
	PCO_2_	20.8 mmHg	35–45 mmHg
	HCO_3_^−^	3.0 mmol/L	22–26 mmol/L
	Base excess	−36.06 mmol/L	−3 to +3 mmol/L
	Lactate	6.5 mmol/L	0.5–2.2 mmol/L
Blood biochemistry	Glucose	16.78 mmol/L	3.9–6.1 mmol/L
	Potassium	6.12 mmol/L	3.5–5.5 mmol/L
	Urea	6.7 mmol/L	1.1–5.9 mmol/L
	Creatinine	41 μmol/L	13–33 μmol/L
	Total bilirubin	25.2 μmol/L	<21 μmol/L
	LDH	416 IU/L	120–250 IU/L
	Uric acid	742 μmol/L	120–320 μmol/L

WBC, white blood cell; CRP, c-reactive protein; LDH, lactate dehydrogenase.

**Figure 2:**
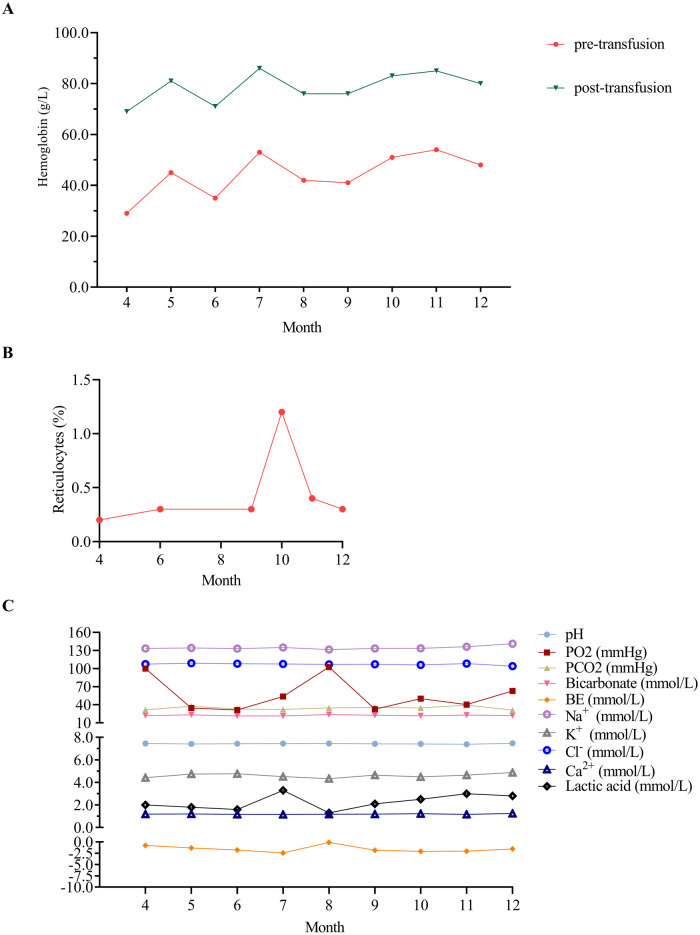
Hematological parameters of the patient during the 1-year outpatient follow-up. **(A)** Hematological profile of the patient before and after transfusion therapy during the 1-year outpatient follow-up. Hemoglobin levels ranged from 29 to 54 g/L (pre-transfusion) to 69–86 g/L (post-transfusion). **(B)** Reticulocytes of the patient during the 1-year outpatient follow-up. **(C)** Blood gas analysis of the patient during the 1-year outpatient follow-up. Blood gas analysis was conducted during outpatient visits at 5, 6, 7, 9, 10, and 11 months using venous blood samples, while arterial blood gas analysis was performed at all other time points. PO_2_, partial pressure of oxygen; PCO_2_, partial pressure of carbon dioxide; BE, base excess.

During the 1-year follow-up period, the patient received RBC transfusions (20 ml/kg) every 4 weeks to maintain hemoglobin levels, which increased from 29 to 54 g/L pre-transfusion to 69–86 g/L post-transfusion. Hemoglobin levels were measured immediately before and after each transfusion (Figure 2A). Corticosteroid was avoided during infancy to mitigate risks of adverse effects on growth and neurodevelopmental issues. The reticulocyte count remained within the range of 0.2%–1.2% (Figure 2B). Symptoms included poor appetite and pale complexion, which improved after transfusions. Blood gas parameters showed no significant abnormalities (Figure 2C). At the 7-month outpatient visit, serum ferritin was elevated at 439.81 μg/L (reference range: 12.00–135.00 μg/L). Other indicators such as total iron-binding capacity, serum iron, transferrin, transferrin saturation, and unsaturated iron-binding capacity unchange. Unfortunately, at 8 months, the child showed growth concerns, particularly short stature (Table 2). The patient is under ongoing follow-up.

**Table 2:** Growth parameters at 8-month follow-up.

Parameter	Percentile	*Z*-score
Weight-for-length	24.3	−0.70
Weight-for-age	6.4	−1.52
Length-for-age	4.3	−1.72

Growth retardation was assessed using three parameters in accordance with WHO Child Growth Standards (https://www.who.int/tools/child-growth-standards).

## Discussion

In our case, a 56-day-old female infant presented with severe anemia and shock, a rare and critical manifestation of DBA. Genetic analysis revealed a *de novo RPS19* mutation, specifically a nucleotide changes of c.3G > T in transcript NM_001022.3, resulting in a heterozygous genotype. To our knowledge, this represents one of the most severe presentations of DBA in infancy, underscoring the importance of early genetic diagnosis and intervention.

A notable feature of our case is the presentation of DBA in a 56-day-old female infant with severe anemia and shock. The infant exhibited a critically low hemoglobin level of 18 g/L, accompanied by severe metabolic acidosis (pH 6.61, base excess −36.06 mmol/L), attributed to a *de novo* mutation in the *RPS19*. To our knowledge, this represents the second young infant reported case of DBA-induced shock in infancy. Smith et al. reported a 7-week-old infant with DBA presenting with respiratory distress, lethargy, and pallor, accompanied by severe anemia (hemoglobin 17 g/L), multi-organ dysfunction, and rhabdomyolysis secondary to shock. Rapid whole genome sequencing identified a *de novo* heterozygous in *RPS26* mutation, and prompt transfusion therapy led to full recovery from acute organ failure (10). Case reports and literature reviews suggest that DBA syndrome results from a *de novo* or sporadic mutation in about two-thirds of cases, while one-third are familial with varying expressivity and penetrance (2). Dashraath et al. documented a rare DBA case with fetal hemoglobin at 21 g/L due to heterozygous deletion on chromosome 3, affecting the *RPL15* gene (5). Moreover, Pelagiadis et al. reported two cases: a 3-month-old male with severe anemia (Hb 18 g/L) from an *RPS26* gene mutation, and a 4-month-old female with worsening pallor (Hb 57 g/L) from an *RPS17* gene mutation (8). These cases collectively highlight the diverse genetic causes and clinical presentations of DBA, emphasizing the need for thorough genetic testing and personalized management strategies, especially in severe infant anemia or shock. To our knowledge, our case represents the youngest reported DBA patient with shock who survived the acute episode.

In this context, shock from severe anemia is likely due to four mains mechanisms. First, the insufficient production of RBC due to a *de novo RPS19* mutation led to severe anemia. The c.3G > T mutation affects the translational start codon of *RPL19* and has not previously been reported in DBA patients. A heterozygous mutation at this position may result in haploinsufficiency of the RPL19 protein. This caused insufficient RBC production, leading to severe anemia and a hemoglobin level as low as 18 g/L, drastically reducing the blood's oxygen—carrying capacity (11). Second, the severe anemia resulted in widespread tissue hypoxia (4). Although the body tried to compensatory by increasingheart rate and cardiac output (12), the efforts were insufficient. As a result, tissues resorted to anaerobic metabolism, producing lactic acid and causing lactic acidosis (13), as evidenced by the infant's pH of 6.61 and a base excess of −36.06 mmol/L. This further impaired cell functions and disrupted the body's acid—base balance. Third, anemia-induced tissue hypoxia and failed compensatory mechanisms led to hypoperfusion. The heart couldn't ensure adequate blood flow, causing circulatory failure (14). The infant's undetectable blood pressure in this case indicated severely compromised cardiac function and ineffective circulation. Fourth, circulatory failure disrupted normal functions, like thermoregulation (15), resulting in hypothermia due to inadequate heat distribution. Fortunately, prompt medical intervention stabilized the infant and prevented critical outcome. This case further underscores the critical importance of early DBA diagnosis to prevent life-threatening complications, such as shock or mortality, arising from delayed intervention.

Shock often termed “oxygen debt,” occurs when oxygen delivery fails to meet metabolic needs (16), especially critical in DBA shock. Immediate correction of severe anemia to restore hemoglobin's oxygen-carrying capacity is vital. However, in emergencies where DBA diagnosis is unclear, start with normal saline volume expansion at 10–20 ml/kg, adjusting based on hemodynamic response. Promptly check hemoglobin levels and immediately administer PRBCs transfusion (10–15 ml/kg) if severe anemia is present to enhance oxygen delivery. Monitor the patient's hemodynamic status and laboratory parameters closely to avoid fluid overload (2, 17). In the present case of a 2.5 kg infant, initial management with 30 ml normal saline followed by PRBCs transfusion and furosemide successfully resolved shock without complications. Although the optimal transfusion threshold for non-hemorrhagic shock in children is unclear, evidence suggests a hemoglobin threshold of <70 to <100 g/L to improve oxygen delivery (16). This is crucial in our case, where the hemoglobin level was 18 g/L, well below the necessary threshold. Restrictive transfusion strategies with the threshold of 60–70 g/L, are not suitable for patients with DBA syndrome due to potential complications. Most patients with DBA require lifelong RBC transfusions to maintain hemoglobin levels for normal growth, development, and quality of life. Typically, receiving 10–15 ml/kg of PRBCs every 3–5 weeks to keep hemoglobin above 80 g/L (1, 4). However, new guidelines suggest aiming for hemoglobin levels of ≥90–100 g/L, tailored to individual needs for optimal outcomes (2). In our case, transfusions of 10 ml/kg resulted in hemoglobin levels of 69–86 g/L (Figure 2A). Based on the latest international guidelines as mentioned above (2), it may be necessary to increase the transfusion volume to 15 ml/kg for this infant.

DBA management involves chronic red blood cell transfusions, corticosteroid therapy, and hematopoietic stem cell transplantation (HSCT) (2, 18). For infants under 1 year, RBC transfusions are preferred over corticosteroids due to growth and neurodevelopment concerns (1, 6, 19). Long-term corticosteroid use can lead to cataracts, osteoporosis, and infections (6). Updated guidelines suggest a prednisone maintenance dose of no more than 0.3 mg/kg per day to reduce toxicity while maintaining effectiveness (2). However, patients unresponsive to corticosteroids may need chronic transfusions or HSCT (20). HSCT is the only curative option for transfusion-dependent DBA patients resistant to glucocorticoid, but it poses risks like graft-vs.-host disease (GVHD) and donor matching difficulties (21). Emerging therapies, such as gene therapy, may significant advance DBA treatment. Preclinical studies using lentiviral vectors to deliver genes like *RPS19*, have shown promise in restoring erythropoiesis and resolving anemia in mouse models (22, 23). While gene therapy hasn't yet been applied to humans with DBA, recent advancements by Voit et al. have enabled clinical trials. Their clinical-grade lentiviral gene therapy with erythroid-specific GATA1 expression improves erythropoiesis in DBA models and patient samples without affecting hematopoietic stem cell function (24). These breakthroughs offer hope for a targeted DBA treatment. Our patient was managed with PRBCs transfusions, as corticosteroid therapy was deferred during infancy due to potential adverse effects on growth and development. Corticosteroid therapy is planned for initiation after the patient reaches 1 year of age, in accordance with current guidelines. HSCT represents a potentially curative option that may be considered if the patient becomes transfusion-dependent. Looking ahead, emerging gene therapy approaches targeting erythroid-specific GATA1 expression show particular promise for *RPS19*-mutated DBA and may provide novel treatment alternatives in the future.

Transfusion is a cornerstone of DBA management (25), but it risks iron overload, leading to serious health issues (1). Effective iron chelation therapy is necessary to address this. Magnetic resonance imaging (MRI) is the preferred method for assessing iron overload in the liver, heart, and pancreas (2, 6). If MRI is unavailable, serum ferritin levels ≥1,000 μg/L and transferrin saturation ≥75% can indicate when to start chelation therapy (6). However, recent guidelines suggest serum ferritin is unreliable for assessing iron overload in DBA (2) due to its inaccuracy and susceptibility to factors like inflammation (26). For those on chronic RBC transfusions, liver iron content should be measured every 12–18 months with chelation therapy considered after 10–20 RBC transfusions (of 10–15 ml/kg), or when MRI shows liver iron concentration ≥6–7 mg/g (6).

Growth concerns in infants with DBA are significant (4). A Chinese study found that 37.98% of 129 children with DBA had short stature (27). Risk factors include female sex, being underweight, cardiovascular malformations, and *RPL11* or *RPS26* mutations (27). Short stature in DBA is linked to disruption in RP synthesis, leading to ribosomal haploinsufficiency. This affects mRNAs translation, impairing cell growth and bone development, and causing growth retardation and short stature (28, 29). Additionally, the genetic heterogeneity of DBA, with mutations in RP genes, results in varied phenotype and incomplete penetrance, including short stature. For example, *RPL11* mutations often cause congenital malformations, and skeletal anomalies, contributing to short stature (30). Genotype-phenotype studies reveal that different DBA mutations lead to varying growth impairment, illustrating the disorder's complexity (31). Furthermore, the p53 pathway, activated by ribosomal stress, plays a role in DBA pathogenesis by causing cell cycle arrest and apoptosis (32, 33), worsening growth defects by impacting growth plate chondrocytes, which are essential for bone elongation and growth. This highlights the intricated nature of growth impairment in DBA (33, 34). Additionally, growth concerns in infants with DBA stem from chronic anemia, which reduces oxygen delivery to tissues essential for development. The body's response to anemia, such as increased cardiac output, can further hinder the growth (35, 36). Moreover, treatments like corticosteroids and blood transfusions also contribute to growth issues; long-term corticosteroid use is known to suppress growth by disrupting hormonal regulation. Additionally, infants with DBA may face nutritional challenges, including increased needs and potential feeding difficulties or dietary restrictions due to treatment side effects (37–40). In the present case, being female, underweight, or having cardiovascular malformations may increase the risk of short stature. The recent international DBA syndrome guideline suggests maintaining pre-transfusion haemoglobinat 90–100 g/L, regardless of age (2). However, our post-transfusion hemoglobin levels were 6.9–8.6 g/L. Further research is needed to determine if this lower hemoglobin target is linked to short stature.

## Conclusion

This case report describes a severe case of DBA in a 56-day-old infant with life-threatening anemia (Hb 18 g/L), metabolic acidosis (pH 6.61), and shock caused by a *de novo RPS19* mutation (c.3G > T). Although prompt transfusions and resuscitation achieved initial stabilization, but the patient developed chronic transfusion dependence (every 4 weeks). This case underscores the critical role of early genetic testing in DBA diagnosis and highlights the need for timely intervention and long-term monitoring to mitigate complications.

## Data Availability

The original contributions presented in the study are included in the article/Supplementary Material, further inquiries can be directed to the corresponding author.
